# The potential of volatile organic compounds to diagnose primary sclerosing cholangitis

**DOI:** 10.1016/j.jhepr.2024.101103

**Published:** 2024-04-27

**Authors:** Robert van Vorstenbosch, Kim van Munster, Georgios Stavropoulos, Daniëlle Pachen, Frederik-Jan van Schooten, Cyriel Ponsioen, Agnieszka Smolinska

**Affiliations:** 1 Department of Pharmacology and Toxicology, NUTRIM School of Nutrition and Translational Research, Maastricht University, Maastricht, The Netherlands; 2 Department of Gastroenterology and Hepatology, Amsterdam University Medical Centres, Academic Medical Center, Amsterdam, The Netherlands

**Keywords:** volatile organic compounds, liver, IBD, biomarkers, PSC, exhaled breath, metabolomics

## Abstract

**Background & Aims:**

Primary sclerosing cholangitis (PSC) is a chronic cholestatic liver disease characterized by progressive inflammation and fibrosis of the bile ducts. PSC is a complex disease of largely unknown aetiology that is strongly associated with inflammatory bowel disease (IBD). Diagnosis, especially at an early stage, is difficult and to date there is no diagnostic biomarker. The present study aimed to assess the diagnostic potential of volatile organic compounds (VOCs) in exhaled breath to detect (early) PSC in an IBD population.

**Methods:**

Breath samples were obtained from 16 patients with PSC alone, 47 with PSC and IBD, and 53 with IBD alone during outpatient clinic visits. Breath sampling was performed using the ReCIVA breath sampler and subsequently analysed by gas chromatography mass spectrometry. Random forest modelling was performed to find discriminatory VOCs and create a predictive model that was tested using an independent test set.

**Results:**

The final model to discriminate patients with PSC, with or without IBD, from patients with IBD alone included twenty VOCs and achieved a sensitivity, specificity, and area under the receiver-operating curve on the test set of 77%, 83%, and 0.84 respectively. Three VOCs (isoprene, 2-octanone and undecane) together correlated significantly with the Amsterdam-Oxford score for PSC disease prognosis. A sensitivity analysis showed stable results across early-stage PSC, including in those with normal alkaline phosphatase levels, as well as further progressed PSC.

**Conclusion:**

The present study demonstrates that exhaled breath can distinguish PSC cases from IBD and has potential as a non-invasive clinical breath test for (early) PSC.

**Impact and implications:**

Primary sclerosing cholangitis is a complex chronic liver disease, which ultimately results in cirrhosis, liver failure, and death. Detection, especially in early disease stages, can be challenging, and therefore therapy typically starts when there is already some irreversible damage. The current study shows that metabolites in exhaled breath, so called volatile organic compounds, hold promise to non-invasively detect primary sclerosing cholangitis, including at early disease stages.

## Highlights

• Volatile organic compounds in exhaled breath can detect primary sclerosing cholangitis in an inflammatory bowel disease population.
• Volatile organic compounds in exhaled breath relate to Amsterdam Oxford score.
• Sensitivity analysis showed stable sensitivity for detection of early-stage PSC.

## Graphical abstract

**Figure:**
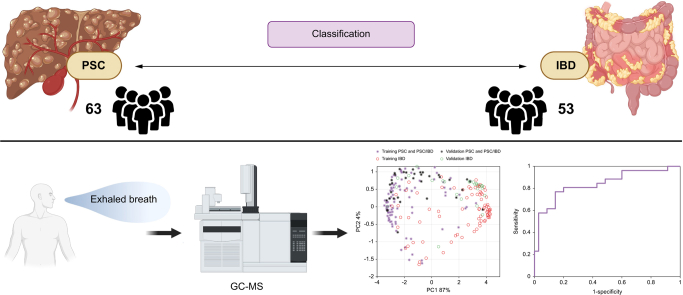

## Introduction

Primary sclerosing cholangitis (PSC) is a chronic cholestatic liver disease characterized by inflammation and fibrosis of both the intrahepatic and extrahepatic bile ducts, leading to formation of multifocal bile duct strictures. Ultimately and commonly, PSC can lead to cirrhosis, liver failure, malignancy, and death.^1^ Its disease aetiology is largely unknown, yet a complex interplay between genetics, exposure and the microbiome is thought to play an important role. One of the hypothesized mechanisms involves a “leaky gut”, which may induce chronic inflammation of the bile ducts.^2^ Roughly 120,000 individuals in the Western world are affected by PSC, although this is likely an underestimation. Currently, the only available curative treatment is liver transplantation, but in 20% of cases the disease re-occurs.^3^

PSC is strongly associated with inflammatory bowel disease (IBD): particularly ulcerative colitis (UC).^4^ Approximately 70% of patients with PSC suffer from IBD, and conversely 5% of patients with IBD have concurrent overt PSC. This mixed phenotype (*i.e.* PSC-IBD) appears different compared to IBD alone,^5^ showing high prevalence of pancolitis and more frequent isolated or predominantly right sided colitis (being rare among classical IBD). It is also associated with a higher incidence of colorectal carcinoma.[6], [7], [8]

The current gold standard to diagnose PSC is imaging via magnetic resonance cholangiography (MRC), which can capture characteristic strictures and/or dilations of either intra- and/or extrahepatic bile ducts.^9^^,^^10^ The disadvantage of MRC is its limited spatial resolution, implying that bile duct lesions must have progressed to macroscopic morphological abnormalities to become detectable. Consequently, MRC oftentimes detects PSC at later stages when anti-inflammatory drugs presumably will be less effective as bile duct fibrosis has already occurred. Moreover, recently Culver *et al.* found MRC features consistent with PSC in 14% of 51 patients with extensive UC and normal liver biochemistry, indicating that the subclinical phase of bile duct injury in UC is present in many more patients than classically diagnosed.^11^ Together this highlights the need for new, ideally non-invasive and cost-effective, diagnostic tools capable of detecting early PSC.^6^^,^^12^

Recently, metabolomics of exhaled breath gained momentum due to its promise to provide such a tool.^9^^,^^10^^,^^13^^,^^14^ Volatile metabolites, or volatile organic compounds (VOCs), reflect the host metabolome and have been related to a variety of disease mechanisms, including oxidative stress, inflammation and microbiome dysbiosis.[14], [15], [16] Previous studies have positively assessed the diagnostic potential of exhaled breath analysis for liver diseases.^14^ PSC, however, has not been investigated by means of exhaled breath; it has only been examined by means of VOC analysis in bile^17^ and urine,^18^ showing promising results. Therefore, the present exploratory *proof of concept* study aims to distinguish PSC with or without IBD from IBD based on (*i.e.* identified) VOCs in exhaled breath using a semi-targeted approach in a study population of patients with PSC, ranging from early mild PSC to end-stage liver disease, and a matched IBD control group.

## Materials and methods

### Patient inclusion

A case-control study was performed among patients with PSC, with or without IBD, compared to those with IBD only. Patients were recruited at the outpatient clinic of the Amsterdam University Medical Center in Amsterdam, The Netherlands, and all participated in the prospective EpiPSC2 registry.^19^ Within this official registry patients are extensively clinically phenotyped, meaning patients with suspicion of secondary sclerosing cholangitis were excluded from this study. The registry contains data on >1,400 patients with PSC; all well-ascertained. The study was approved by the institutional review board of the Amsterdam University Medical Centre (NL64879.018.18) and all patients provided written informed consent.

IBD controls were matched 1:1 based on sex, age, IBD type and colectomy status. Inclusion criteria for PSC was an established diagnosis based on the EASL criteria^20^ and for IBD an established IBD diagnosis based on ECCO criteria.^21^ A diagnosis of PSC only (without IBD) could only be made in case of a normal ileocolonoscopy in the previous 5 years.

Other inclusion criteria were an age range above 18 years old, and a BMI range from 19 to 30. Exclusion criteria for all three groups were: unable to provide informed consent, the presence of any disease that compromises the immune system such as HIV positive or organ transplantation, the presence of any other liver disease, the presence of active or untreated tuberculosis, and the use of chemotherapy agents. Patients with IBD were excluded in case of abnormality in liver tests such as elevated alkaline phosphatase or transaminases.

### Sampling and data acquisition

Breath and blood samples were acquired during outpatient clinic visits in a dedicated room. For the breath sampling, the ReCIVA (Owlstone Medical, Cambridge, UK) breath sampler connected to a CASPER air pump (Owlstone Medical, Cambridge, UK) was used.^22^ Briefly, the CASPER lowers background VOC levels by filtering ambient air before passing it on to the ReCIVA and allowing comfortable breathing for participants. ReCIVA traps only the relevant fraction of exhaled breath (*i.e.* the alveolar fraction). Each participant was requested to breathe for approximately 5 min in the device, trapping the volatiles *in duplo* onto stainless steel carbon-filled sorption tubes (Tenax/Carbograph-5TD TD tubes [Markes International Ltd, Llantrisant, UK]).^22^ Two hours before sampling, the participants were asked to abstain from eating and drinking any liquids, beside water. All samples were stored at 5 °C until subsequent chemical analysis.

Upon storage, the breath samples were analysed using thermal desorption gas chromatography-*time of flight*-mass spectrometry (GC-*tof*-MS). In short, VOCs are separated via GC, and then identified via *tof*-MS. The experimental settings of the GC-*tof*-MS are described elsewhere,^23^ and the VOC identification was achieved by using the NIST Mass Spectral Search Program v2.3. An internal standard (*i.e.* Bromobenzene-D5) was injected into every sample before measuring and quality controls (SUPELCO Analytical; reference number 44589) were run in between breath samples throughout the GC-*tof*-MS runs to monitor and control instrument performance.^9^

Participants donated blood as part of their regular blood testing procedure. These blood samples were used to measure thrombocytes, alkaline phosphatase, aspartate aminotransferase, bilirubin and alanine aminotransferase. Based on these parameters the prognostic Amsterdam-Oxford score was calculated for PSC cases. The Amsterdam-Oxford scoring system was used as a proxy to estimate disease stage in terms of early *vs*. advanced, and predict disease outcomes. Lastly, patients also filled in a questionnaire regarding BMI, smoking status, whether a specific diet is followed (*i.e.* meat-, lactose-, gluten- or carbohydrate-free diet), use of supplements and medication. Demographic data were recorded from medical charts. Smoking status was defined as yes (current smoking or smoking in the past 2 years) or no (never smoking or stopped more than 2 years before). To assess PSC symptom severity all patients with PSC completed the simple cholestatic complaints score.^24^ To assess IBD severity, all patients with UC completed the simple clinical colitis activity index (SCCAI),^25^ all patients with Crohn’s disease completed the Harvey Bradshaw index (HBI).^26^ In addition, calprotectin was measured in patients with IBD who additionally provided fecal samples (n = 37). Fibroscan was conducted in all patients with PSC on the same day as breath sampling.^27^

### Data handling and statistical modelling

The present study followed a *semi-targeted* approach. In contrast to an untargeted analysis, where as many VOCs are quantified as possible, a semi-targeted analysis considers only an *a priori* defined set of VOCs. Such a reduced feature set results in more reliable VOC quantities (*i.e.* increased signal to noise ratio), in reduced chances of creating an overfitted statistical predictive model, and therefore in increased reproducibility of the results. Moreover, it directs the statistical model to a biologically interpretable model. The semi-targeted set was based on VOCs that have previously been reported in the literature to be related to liver impairment^14^ (Box 1). A few VOCs, including aldehydes and alkanes that relate to oxidative stress and lipid peroxidation, both implicated in PSC pathophysiology, were added to this list.

Box 1 **Compounds selected as possible targets to distinguish PSC±IBD from IBD alone.**

**Table:** 

Acetaldehyde	Pentanal	Decane
Ethanol	2-Pentanone	Octanal
Acetone	Hexanal	Limonene
Pentane	Octane	Undecane
Isoprene	Nonane	Nonanal
2-Propanol	Styrene	Dodecane
Dimethyl-sulphide	2-Nonene	Decanal
Carbon-disulphide	2-Octanone	Tridecane
Butanal	Heptanal	Indole
2-Butanone	Beta-pinene	Undecanal
Hexane	Alpha-pinene	
Benzene	Benzaldehyde

IBD, inflammatory bowel disease; PSC, primary sclerosing cholangitis.

Alt-text: Box 1

The selected compound peaks were integrated using their characteristic mass fractions. Next, the data were normalised by the internal standard and log transformed to account for data heteroscedasticity and skewness.^28^

The statistical modelling process included data exploration via unsupervised random forest (RF)^29^ to assess the presence of any groupings in the data. Next, classification was performed using RF.^30^ Here, first the data was split into a training (*i.e.* 80% of the data) and independent test (*i.e.* the remaining 20% of the data) set using the Kennard and Stone algorithm.^31^ Samples coming from the same patient were always kept together in either the training or the test set to avoid overestimation of the model. The training set was used to build the RF model (*i.e.* PSC and PSC/IBD *vs.* IBD) and the independent test served only to test its classification performance. The RF model parameters (*i.e.* number of trees, predictors, number of splits per tree and samples per tree terminal leaf per RF model), as well as to the number of VOCs to be kept for the final classification model were optimized within a 1,000-iteration loop. A detailed description of how this 1,000-iteration loop is performed is described elsewhere.^32^ In the present study, 1,000 trees per model were used. Optimization resulted in a minimum number of samples in a terminal leaf of six and five splits per tree maximum. The VOCs that appeared as important in at least 40% of iterations were kept for further analysis and tested for significance using the Wilcoxon signed rank test. Model performance was assessed using sensitivity, specificity, and area under the receiver-operating characteristic curve (AUC). Additionally, a sensitivity analysis was carried out by comparing model performance of patients belonging to the 50% and 25% lowest to the respective highest Amsterdam-Oxford score groups. Sensitivity analysis also included investigating the effect on the model of PSC-only patients, as their phenotype is not identical to the PSC/IBD phenotype. Moreover, correlating patterns were investigated between the selected VOCs and the Amsterdam-Oxford score. Note that for this analysis (and its validation), a separate test set was created using the Duplex algorithm (*i.e.* 33% of samples in the test set). In addition, a sensitivity analysis of the VOC-based model to classify patients with PSC and normal values of alkaline phosphatase (*i.e.* <120 U/L) was performed. The final analysis concerned the influence of potential confounders (*i.e.* smoking, diet, medication, age, supplements, gender, IBD-activity and colectomy) on the exhaled breath profile using regularised multivariate analysis of variance (rMANOVA).^33^

Peak area integrations were performed in Xcalibur v2.2 SP1.48 software. Subsequent data analyses were performed using MatLab R2016b version.

## Results

### VOC analysis

In total, 16 patients with PSC alone, 47 with PSC with IBD, and 53 with IBD alone were sampled and included in the study analysis. Demographic and patient characteristics are summarised in Table 1. PSC was diagnosed using MRC in most patients, but (diagnostic) endoscopic retrograde cholangiography was used instead in some patients diagnosed long ago. Liver biopsy to confirm the diagnosis of PSC or to diagnose small duct PSC was performed in 20/58 (34%) patients. To increase and balance sample sizes between groups, the PSC and PSC/IBD patient populations were fused into one class and compared to IBD alone. In PSC-only patients the latest normal ileocolonoscopy was on average 21 months before breath sampling, with a minimum of 2 and a maximum of 42 months. UDCA was not used in patients with IBD only, while biological use was significantly higher in this group. IBD disease activity, based on HBI or SCCAI, did not significantly differ between patients with IBD only and PSC-IBD (1 *vs.* 1, n.s.). One patient with IBD and two with PSC-IBD used metronidazole ovules once weekly. And one patient with PSC-IBD used rifaximin daily. No other current or recent antibiotic use was reported.

**Table 1:** Patient characteristics.

	PSC (±IBD) n = 63	IBD n = 53	*p* value
Age^1^	48	47	0.61
Large duct PSC	55 (87%)	—	NA
PSC disease duration, years^2^	8.5 (1-35)	—	NA
IBD	47 (75%)	53 (100%)	<0.01
Ulcerative colitis	31 (66%)^3^	36 (68%)	0.65
Colectomy before inclusion	15 (32%)^3^	9 (17%)	0.10
Male sex	40 (63%)	28 (53%)	0.17
Smoking	1 (%)	4 (%)	0.13
Alcohol, glasses per week^2^	0 (0-7)	1 (0-7)	0.03
Any specific diet	4 (6%)	6 (11%)	0.26
Alkaline phosphatase *(tULN)*^2^	1.1 (0.4-4.7)	0.7 (0.3-0.9)	*<0.01*
AST *(tULN)*^2^	0.8 (0.4-4.7)	0.6 (0.4-1.8)	<0.01
ALT *(tULN)*^2^	0.9 (0.4-9.0)	0.6 (0.3-1.8)	<0.01
Bilirubin *(tULN)*^2^	0.7 (0.2-3.0)	0.8 (0.3-2.2)	0.62
Amsterdam-Oxford Score 2	1.4 (0.5-4.4)	—	NA
MELD-score 2	6 (6-26)	—	NA
Fibroscan F0-1	41 (71%)	—	NA
Fibroscan F2-3	7 (12%)	—	NA
Fibroscan F4	10 (17%)	—	NA
SCCS sum score 2	1 (0-6)	—	NA
SCCS pruritus score 2	0 (0-3)	—	NA
HBI or SCCAI score^*,*^	1 (0-9) 3	1 (0-14)	0.34
Medication use			
Ursodeoxycholic acid	46 (73%)	0 (0%)	<0.01
Corticosteroids	6 (10%)	4 (8%)	0.27
Thiopurines	3 (5%)	4 (8%)	0.41
Biologicals	4 (8%)	18 (29%)	<0.01

ALT, alanine aminotransferase; AST, aspartate aminotransferase; HBI, Harvey Bradshaw index; tULN, times upper limit of normal; SCCAI, simple clinical colitis activity score; SCCS, simple cholestatic complaints score. Statistical test used: ANOVA and Fisher exact test.

1 Mean (SD).

2 Median (range).

3 Data based on patients with PSC-IBD only.

Each patient was sampled in at minimum duplicate, resulting in 234 measurements. Of these, 173 measurements (93 PSC and PSC/IBD and 80 IBD) were used as the training set, and the remaining 61 (35 PSC and PSC/IBD and 26 IBD) were used as the independent internal test set. The most discriminatory compounds used for building the final classification RF model were 20 (Box 2). Eight compounds were distributed significantly different between groups: acetone, hexanal, octane, 2-octanone, decane, undecane, dodecane, and decanal. The concentration of acetone and hexanal increased in PSC cases, whereas the concentration of the other six compounds decreased.

Box 2 **Overview of the volatile organic compounds found to be important in the 1,000-iteration procedure for building the final RF classification model.**

**Table:** 

Ethanol	↑ Hexanal∗	Limonene
↑ Acetone∗	↓ Octane∗	↓ Undecane∗
Pentane	Nonane	↓ Dodecane∗
Isoprene	↓ 2-Octanone∗	↓ Decanal∗
Carbon-disulphide	Alpha-pinene	Tridecane
Pentanal	Benzaldehyde	Undecanal
2-Pentanone	↓ Decane∗

∗Abundance is significantly different between PSC±IBD and IBD alone; The arrow indicates whether their relative abundance is increased or decreased in the breath of PSC ± IBD group when compared to IBD. IBD, inflammatory bowel disease; PSC, primary sclerosing cholangitis; RF, random forest.

Alt-text: Box 2

**Fig. 1:**
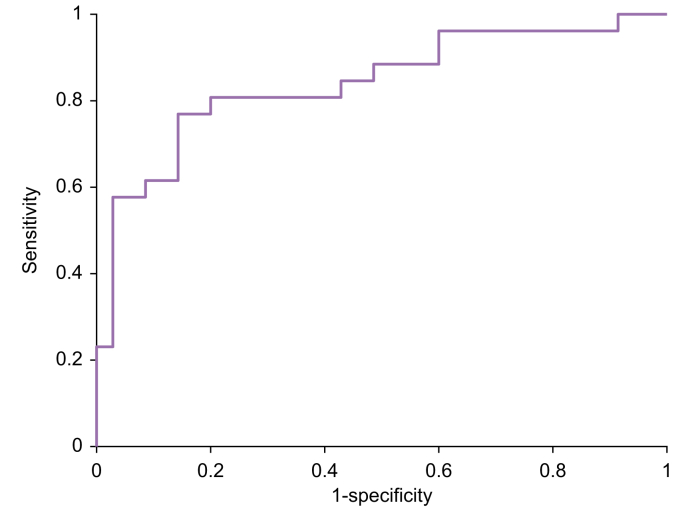
Receiver-operating characteristic curve of the independent test set. AUC = 0.84, sensitivity = 77%, and specificity = 83%.

**Fig. 2:**
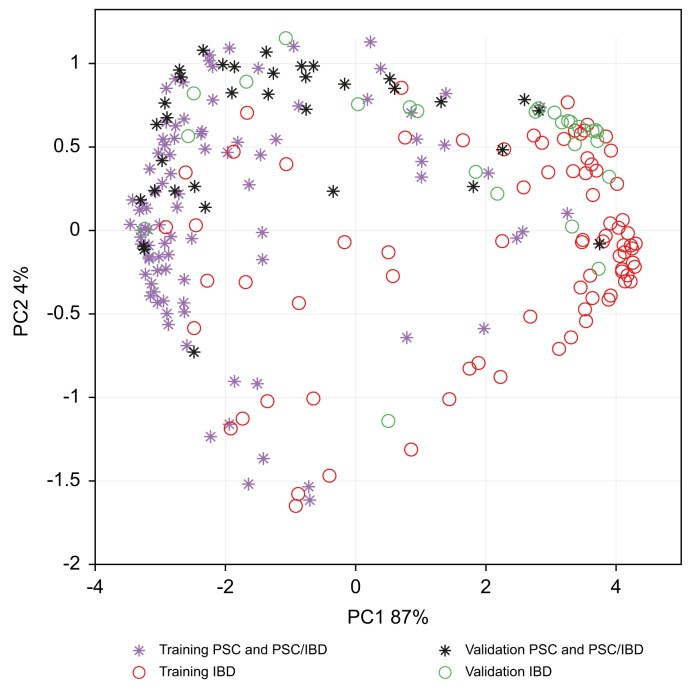
Score plot based on the proximities of the training and independent test sets.

Exploratory analysis of the dataset via unsupervised RF did not show any underlying groupings in the data (*i.e.* batch effects, results not shown), whereas the final RF predictive model demonstrated a classification performance by achieving a sensitivity of 77%, a specificity of 83%, and an AUC of 0.84 for the independent test set (Fig. 1). The precision of the model is further demonstrated by the clear separation between both groups, as shown in the principal coordinate scores according to the RF model (Fig. 2). Subgroupings based on PSC type (*i.e.* small or large) were not observed, but the current dataset was underpowered to assess differences using supervised strategies (*i.e.* n = 8 for small type PSC). Moreover, investigation of model performance upon leaving out PSC-only cases resulted in an AUC of 0.83, thereby showing its impartiality towards this group. A further sensitivity analysis compared the 50% and 25% highest as well as lowest Amsterdam-Oxford-scored patients to each other. The lowest 25% of Amsterdam-Oxford scores corresponds with a predicted median transplant-free survival of >95% after 5 years and >79% after 20 years. Patients in the highest IQR had a median transplant-free survival of <89% after 5 years and <62% after 20 years. AUCs were stable across these comparisons with values of 0.87 and 0.82 for the highest and lowest 50%, and 0.83 and 0.82 for the highest and lowest 25% Amsterdam-Oxford-scored patients, respectively.

### VOCs in comparison to Amsterdam-Oxford score

Upon building the RF classification model, the twenty selected markers were further tested to correlate to the Amsterdam-Oxford score. Here, testing the trained RF model resulted in a correlation coefficient of R = 0.65 (*p =* 0.02) on the independent test set, where isoprene, 2-octanone and undecane were selected as the prognostic volatiles.

### VOCs and normal alkaline phosphatase

**Fig. 3:**
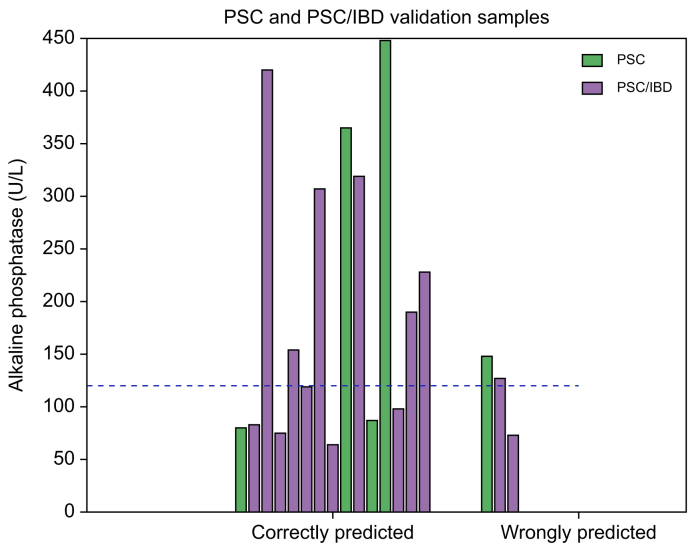
Visualization of correct *vs.* incorrect predicted breath profiles and alkaline phosphatase values above or below the upper limit of normal of 120 U/L. Sensitivity of PSC breath profile in patients with a normal alkaline phosphatase = 87.5%. IBD, inflammatory bowel disease; PSC, primary sclerosing cholangitis.

Since patients with PSC can have normal levels of alkaline phosphate, especially in early-stage disease, it is relevant to obtain a model that is capable of properly predicting patients with normal levels of this blood parameter. A sensitivity analysis of the PSC and PSC/IBD individuals with normal levels of alkaline phosphate (*i.e.* <120 U/L) showed that the VOC profile correctly predicted samples with or without PSC with a sensitivity of 87.5% (Fig. 3).

### Confounding factor analysis

The possibility of confounding factors causing optimistic study outcomes was examined. No baseline characteristics were significantly different between the two classes except for the ursodeoxycholic acid and biologicals medications, which were taken by a majority of patients with PSC and IBD, respectively. Within the respective groups we tested the influence of these medications on the overall volatile profile of the selected markers between those that did and those that did not take this medication using rMANOVA. Upon multivariate analysis no significant effects of these medications on the selected markers were found (*p =* 0.73 for ursodeoxycholic acid and *p =* 0.25 for the biologicals). Other possible confounders that were tested were IBD-activity (*i.e.* through HBI and SCCAI, or through calprotectin levels) and colectomy. No statistical influence was observed for either (*i.e.* HBI & SCCAI: *p =* 0.6, calprotectin: *p =* 0.59, and colectomy: *p =* 0.12).

## Discussion

The present study investigated the potential clinical application of the exhaled breath in identifying PSC cases amongst IBD cases. A breath VOC profile was able to discriminate between PSC and IBD cases. Moreover, the three VOCs isoprene, 2-octanone and undecane correlated to the prognostic Amsterdam-Oxford score. A breath profile of 20 VOCs (Box 2) was able to identify PSC cases amongst IBD cases with a sensitivity of 77%, a specificity of 83% and an AUC of 0.84 for an independent test set (Fig. 1, Fig. 2). Sensitivity analysis showed that the VOC-based classification model performed equally well in patients with normal alkaline phosphatase and the 25% lowest Amsterdam-Oxford scores compared to the whole group. Hence, the observed breath VOC profile holds promise as a screening tool for PSC in IBD cases, potentially leading to detection of early-stage PSC. It must be acknowledged, however, that these numbers are still inferior to MRC (*i.e.* sensitivity and specificity of 86% and 94%, respectively). Therefore, it should be underlined that the potential added value of such a breath test would be its potential for early-stage diagnosis. Recent preliminary studies demonstrate that exhaled breath signals can be further enhanced by following liver metabolism of substrates administered to the patient (*i.e.* so called EVOC probes).^34^ By following a multitude of such substrates multiple different hepatic metabolic pathways can be investigated. Therefore, exhaled breath analysis is already being further enhanced and might already carry more potential than well-established blood tests that lack early-stage sensitivity.^35^ A sensitivity analysis of a VOC-based classification model indicated that the model can correctly predict PSC in patients with normal values of alkaline phosphate with sensitivity of 87.5%. This indicates that a VOC-based model and the observed breath VOC profile might potentially serve as a more specific or robust screening tool than alkaline phosphatase.

The majority of the 20 VOCs identified in the present study can be categorised into four main categories: alkanes, alkenes, ketones, and aldehydes. Since this selection resulted from a semi-targeted analysis based on current literature, the VOCs validate their importance as concluded by previous studies.^14^ Of the selected VOCs, limonene is known as exogenous VOC, originating from foods and drinks. It is metabolised by the P450 enzymes CYP2C9 and CYP2C19 into other compounds such as perillyl alcohol, trans-isoperitenol, and trans-carveol.^14^ In liver impairment, these enzymes are reduced, resulting in reduced limonene clearance from the body. As such, limonene may be a marker of generally impaired liver function, which will be more pronounced with ongoing deterioration. Many of the selected VOCs selected in the current study relate to lipid peroxidation, a process triggered by increased inflammation generating alkanes and aldehydes, that can be converted into alcohols or ketones by CYPs or aldo-keto reductases, respectively.^14^ Moreover, increased concentrations of alkanes and aldehydes have previously been linked to IBD.^8^ The increased acetone concentrations found in the present study might originate from increased hepatic insulin resistance, resulting in increased levels of triglycerides, free fatty acids, and ketones. Interestingly, isoprene, 2-octanone and undecane correlated with disease prognosis, further validating their relevance in PSC.

Notably, some VOCs were not selected by the current model. Disregarding dimethyl-sulphide was striking, since it had repeatedly been selected in previous studies and its origin is linked to incomplete metabolism of sulphur-containing amino acids in the transamination pathway. Finally, another VOC that should be critically assessed is isoprene. According to the literature its origin is conflicting, questioning its value as a potential biomarker. Isoprene can originate from impairment in the cholesterol biosynthesis pathway, it can be the result of disturbed colon flora, or it can be the result of exercise.^14^ In the present study participants were prevented from exercising 2 h before sampling; however, this does not fully exclude the fact that isoprene might have originated from exercising since it can be stored in muscle cells to be released later.^36^

The present study demonstrates to the best of our knowledge for the first time that exhaled breath can be a potential and possibly early biomarker for diagnosing PSC in IBD. A strength of the present study was that all the participants were sampled at the same location, using the same background-reducing equipment, and by the same personnel. Moreover, GC-MS instrument performance was monitored using quality control samples in regular intervals throughout the measuring process. The effects of the remaining factors (*i.e.* age, gender, smoking, diet, medication, and supplements) were investigated using rMANOVA. No significant differences were found for any of the aforementioned possible confounding factors. Another strength was its semi-targeted approach, thereby focusing only on and thus validating previously reported and biologically interpretable VOCs. Lastly, an independent test set was used to test model predictions. To date, most of the exhaled breath VOC analyses that have been conducted in liver diseases were not independently validated due to their small sample size or they did not account for various confounding factors that could have influenced their results.^14^ Some limitations have to be addressed. First, although all patients with PSC were extensively clinically phenotyped using an official registry and EASL guidelines, genotyping was not performed. Therefore, it is possible that disease varieties were included that fall outside the scope of the current study. Second, although we corrected for type of diet, use of supplements and medications including over the counter drugs, it cannot be fully excluded that there was an imbalance in these factors between groups, as we did not prescribe a fixed diet prior to the measurements. Although we excluded IBD cases with increased liver tests, according to previous observations in patients with UC from the UK,^11^ the IBD group could have included early-stage PSC cases in up to 10%, thereby potentially somewhat biasing the comparator group. However, this would infer an underestimation of the predictive abilities of the VOC profile. Another point to raise is that as a consequence of the semi-targeted approach, it is possible that potentially even more discriminating VOCs were not included in the current study. However, for now we underline that the biological interpretation of such novel VOCs would have been extremely challenging. Further research is needed to look into these unknowns. Additionally, the current study used the Amsterdam-Oxford score as a proxy for disease staging. However, it must be stated that based on the endpoints this scoring system was developed for (*i.e.* long-term transplant-free survival probabilities), *high-* and *low-risk* would perhaps be more appropriate terms in contrast to early and late stage. Other scoring systems, including the Mayo risk score, are equally troublesome, as they were developed to assess 4-year mortality risk of patients with end-stage disease. This challenge in defining early-stage PSC highlights the need for more research towards early-stage diagnosis of PSC. In the current study we committed to the phrase *early stage*, as *low-risk PSC* could be misinterpreted as patients with IBD without PSC at low risk of developing PSC. Other phrases could include well-managed *vs*. critical-stage PSC, but those similarly can raise confusion. Lastly, by focusing only on PSC in the current proof of concept study, it is unknown if the selected markers are specific to PSC or indicative of other liver diseases.

In conclusion, the present study demonstrates the diagnostic potential of exhaled breath analysis to detect PSC in IBD cohorts. Our findings warrant further research into the clinical application of VOCs towards PSC detection and monitoring, which ultimately may lead to a diagnostic test for early-stage PSC. Potentially this may enable pre-emptive treatment in patients with IBD at risk of developing overt biliary disease.

## Abbreviations

GC-*tof*-MS, gas chromatography-*time of flight*-mass spectrometry; HBI, Harvey Bradshaw index; IBD, inflammatory bowel disease; MRC, magnetic resonance cholangiography; PSC, primary sclerosing cholangitis; rMANOVA, regularised multivariate analysis of variance; RF, random forest; SCCAI, simple clinical colitis activity index; SCCS, simple cholestatic complaints score; UC, ulcerative colitis; VOCs, volatile organic compounds.

## Financial support

The present study was supported by the VENI grant, Netherlands organization for scientific research (NWO) no. 016 VENI 178.064.

## Conflict of interest

The authors declare that they have no competing financial interests or personal relationships that could appear to influence the work presented in this paper. AS is an advisor at Owlstone Medical.

Please refer to the accompanying ICMJE disclosure forms for further details.

## Authors’ contributions

RvV: Data Curation, Formal Analysis, Investigation, Validation, Visualization, Writing – Original Draft; KvM: Data Curation, Investigation, Resources, Writing – Original Draft; GS: Data Curation, Formal Analysis, Investigation, Visualization; DP: Formal Analysis, Data Curation, Methodology; FJvS: Conceptualization, Funding Acquisition, Methodology, Review & Editing; CP: Conceptualization, Methodology, Resources, Supervision, Review and Editing; AS: Conceptualization, Funding Acquisition, Investigation, Supervision, Review and Editing.

## Data availability statement

In light of patient privacy, data can be made available only upon reasonable request.
